# Machine Learning-Based Biomarker Discovery from Serum Trace Elements and Biochemical Parameters in Patients with Nasal Polyps

**DOI:** 10.1007/s12011-025-04718-7

**Published:** 2025-06-23

**Authors:** Berrin Aydin, Omer Faruk Kocak, Saime Ozbek Sebin, Fatma Betul Ozgeris

**Affiliations:** 1 Department of Otorhinolaryngology, Erzurum City Hospital, Erzurum, Turkey; 2 Department of Chemical Technology, Vocational School of Technical Sciences, Ataturk University, Erzurum, 25240 Turkey; 3 Department of Physiology, Faculty of Medicine, Atatürk University, Erzurum, Turkey; 4 Department of Nutrition and Dietetics, Faculty of Healthy Sciences, Ataturk University, Erzurum, Turkey

**Keywords:** Chronic rhinosinusitis, ICP-MS, Serum metals, Oxidative stress, Multivariate analysis

## Abstract

Nasal polyps (NP) are benign mucosal outgrowths associated with chronic inflammation that can significantly reduce quality of life. This study aimed to evaluate changes in inflammation, oxidative stress, and trace element homeostasis in NP patients and to identify potential non-invasive diagnostic biomarkers. A total of 22 patients with NP and 19 healthy individuals were included in the study. Serum levels of trace elements, including zinc (Zn), copper (Cu), and selenium (Se), were measured using inductively coupled plasma mass spectrometry (ICP-MS). Biochemical parameters including white blood cell count (WBC), red blood cell count (RBC), platelet count (PLT), eosinophils (EO), hemoglobin (HGB), glucose, creatinine, alanine aminotransferase (ALT), and thyroid-stimulating hormone (TSH) were assessed, along with inflammatory indices such as neutrophil-to-lymphocyte ratio (NLR) and platelet-to-lymphocyte ratio (PLR). Data were analyzed using classical statistical methods, including the Shapiro–Wilk test, independent samples *t*-test, Mann–Whitney *U* test, and receiver operating characteristic (ROC) analysis. Multivariate analyses such as principal component analysis (PCA), orthogonal partial least squares discriminant analysis (OPLS-DA), and variable importance in projection (VIP) scoring were performed. In addition, machine learning algorithms including Naive Bayes, support vector machines (SVM), random forest, k-nearest neighbors (KNN), and logistic regression were employed. SHapley Additive exPlanations (SHAP) analysis was used to interpret the most influential features of the best-performing model. Compared to controls, NP patients exhibited significantly higher levels of WBC, Cu, glucose, and NLR along with significantly lower levels of Zn, PLR and the Zn/Cu ratio. Specifically, the mean Zn level was 2130.974 ± 3516.317 µg/mL in the NP group versus 11,331.127 ± 27,697.378 µg/mL in controls (*p* = 0.018). Cu (AUC = 0.866), glucose (AUC = 0.777), and WBC (AUC = 0.748) showed strong discriminative power. OPLS-DA revealed clear group separation, highlighting Cu, Zn/Cu, glucose, Se, and PLR as high-impact variables. Optimized logistic regression achieved 100% classification accuracy, with SHAP analysis confirming Zn, Zn/Cu, Cu, and glucose as the most influential features. These preliminary findings suggest that inflammation, trace element imbalance, and metabolic alterations can be detected biochemically in NP patients. Parameters such as serum Zn and Cu levels, Zn/Cu ratio, glucose, and inflammatory indices may serve as promising non-invasive diagnostic biomarkers. Further validation in larger and independent cohorts is warranted before clinical implementation.

## Introduction

Nasal polyps (NP) are benign mucosal growths that cause significant clinical problems such as nasal obstruction, loss of sense of smell, headache and chronic rhinosinusitis, with a prevalence ranging from 1 to 4% in the general population, develop as a result of chronic inflammation of the nasal cavity and paranasal sinuses, and seriously reduce the quality of life [1, 2]. Although the etiology of NP is not known exactly, it is thought that chronic inflammation, immunological imbalances, oxidative stress and tissue remodeling processes may be effective in the pathogenesis of the disease [3, 4].

Polyps are thought to be a multifactorial disease that may be caused by factors such as low immunity, fungi, superantigens, biofilms, atopy, and mucociliary dysfunction [5, 6]. However, it has been specifically stated that the most important factor in the development of NP is inflammation [7, 8]. In this context, it is thought that routine biochemical parameters may be important in NP disease. While eosinophils (EO) counts are associated with the severity of inflammation in the evaluation of systemic inflammation in nasal polyps [9], changes in red blood cell count (RBC) and hemoglobin (HGB) levels have been associated with the systemic effects of chronic inflammation [10]. It has been reported that inflammatory indices such as neutrophil-to-lymphocyte ratio (NLR) and platelet-to-lymphocyte ratio (PLR) are extremely important in diagnosis and prognosis [11]. Although such biochemical parameters are of critical importance in understanding the systemic effects of the disease, it is understood that they are not sufficient for comprehensive evaluation of the complex pathophysiology of NP.

In recent years, the role of trace elements in the pathogenesis of chronic inflammation and immunological diseases has become a popular research topic. Trace elements are essential micronutrients that play crucial roles in various physiological processes, including immune function, antioxidant defense, and inflammatory responses [12, 13]. Zinc (Zn) plays a critical role in the regulation of immune functions with its effects of supporting the immune system, reducing inflammation and enhancing antioxidant defense [12]. In contrast, copper (Cu) can exhibit proinflammatory effects at excessive levels, contributing to increased oxidative stress and exacerbation of inflammation [13]. Selenium (Se), with its strong antioxidant capacity, has an inflammation-reducing effect as a cofactor of antioxidant enzymes such as glutathione peroxidase; selenium deficiency can trigger the formation of chronic diseases by increasing inflammation and oxidative stress [14].

Environmental factors, especially in rapidly urbanizing and industrial regions such as Turkey, can significantly affect trace element exposure and accumulation in human tissues. Recent studies have shown that heavy metals and trace elements, including lead and manganese, accumulate at higher levels in the nasal tissues of individuals living in urban areas compared to those in rural regions, primarily due to increased environmental pollution. Given this context, the investigation of trace elements in nasal polyp patients in our region is of particular relevance [15]. Okur et al. (2013) specifically demonstrated that Zn and Se levels were significantly lower in nasal polyp tissues compared to normal nasal mucosa, suggesting their potential role in the development of NP [16]. This regional context provides a strong rationale for investigating trace element levels in nasal polyp patients in our geographic area.

The relationship between trace elements and biochemical parameters in nasal polyp patients is complex and multifaceted. Zn deficiency has been shown to impair immune function and increase susceptibility to inflammatory conditions, potentially exacerbating the inflammatory processes observed in nasal polyps [17]. Se, through its role in glutathione peroxidase activity, directly influences oxidative stress markers and may modulate the inflammatory response in nasal tissues [16]. Cu, while essential for various enzymatic functions, can contribute to oxidative damage when present in excess, potentially affecting red blood cell parameters and inflammatory indices [13, 18]. Nikakhlagh et al. (2021) demonstrated that tissue Zn levels were significantly lower in polyp tissues compared to healthy nasal mucosa, while Se showed no significant difference, highlighting the differential roles these elements may play in nasal polyp pathophysiology [5].

However, the number of studies on serum trace element levels in patients with NP is limited [19], and the relationship between these elements and routine biochemical parameters remains poorly understood. This knowledge gap presents an important opportunity for research that could enhance our understanding of nasal polyp pathogenesis and potentially identify new therapeutic targets.

In addition to classical statistical methods, the use of machine learning and multivariate analysis techniques is increasingly prevalent in clinical research to uncover complex data patterns. Machine learning methods such as principal component analysis (PCA), orthogonal partial least squares discriminant analysis (OPLS-DA), and various classification algorithms (logistic regression, support vector machines (SVM), Random Forest, Naive Bayes, and k-nearest neighbors (KNN)) can outperform traditional approaches in biomarker identification, classification, and improving clinical diagnostic accuracy [20–22]. These advanced analytical techniques are particularly valuable for investigating the complex interrelationships between trace elements and biochemical parameters, as they can identify patterns and associations that might be missed by conventional statistical approaches [23]. However, studies in the literature on the use of machine learning algorithms for the evaluation of NP remain quite limited, representing a significant gap in current research.

In light of this information, our study aimed to evaluate serum Zn, Cu and Se levels in patients with NP, as well as biochemical parameters such as white blood cell count (WBC), RBC, platelet count (PLT), EO, HGB, Glucose, creatinine, alanine aminotransferase (ALT), and thyroid-stimulating hormone (TSH), NLR and PLR. The selection of these specific trace elements was based on their established roles in inflammatory processes, their environmental relevance in our region, and the limited existing research on their relationship with nasal polyp pathophysiology. In addition to classical statistical analyses, the study also aimed to identify clinically significant biomarkers in NP, evaluate their classification performance and contribute to a clearer understanding of the pathogenesis of the disease by using PCA, OPLS-DA and various machine learning algorithms. By integrating trace element analysis with advanced computational methods, this research seeks to provide new insights into the complex interplay between environmental factors, trace element status, and biochemical parameters in NP development, potentially leading to improved diagnostic and therapeutic approaches for this challenging condition.

## Materials and Methods

### Study Design and Participants

This study included 22 patients diagnosed with NP, aged between 18 and 75, who applied to the Otolaryngology Department of Erzurum City Hospital, and 19 healthy volunteers with a similar distribution in terms of age and gender, without any chronic diseases. Exclusion criteria were the presence of systemic inflammatory disease, malignancy, and a recent history of infection. The study was conducted between April and October 2024. Patients were selected consecutively from individuals applying to the Otolaryngology Department during this period, based on clinical diagnosis and eligibility criteria. Healthy volunteers were recruited from hospital staff and individuals undergoing routine check-ups, matched for age and sex distribution. All participants were from Erzurum and surrounding districts, ensuring geographical consistency in environmental exposure.

This study was conducted with the approval of the Atatürk University Faculty of Medicine Ethics Committee numbered B.30.2.ATA.0.01.00/638, and written informed consent was obtained from all participants.

### Biochemical and Clinical Data Collection

Blood samples were collected from patients after an overnight fast. Following venipuncture, part of each blood sample was sent to the hospital laboratory for routine biochemical analysis. The remaining portion, intended for trace element analysis, was transported to the Ataturk University Eastern Anatolian High Technology Research and Application Center (DAYTAM). These samples were centrifuged within 2 h of collection at 3000 rpm for 10 min to separate the serum. The obtained serum samples were then aliquoted and stored at –80 °C until analysis. Serum Zn, Cu and Se concentrations, age, gender, accompanying characteristics and biochemical parameters (WBC, RBC, PLT, EO, HGB, Glucose, creatinine, ALT, TSH, NLR, PLR) of each patient were recorded. NLR and PLR ratios were derived by calculating the neutrophil/lymphocyte and platelet/lymphocyte ratios [24].

### Trace Element Analysis

In determining trace element (Zn, Cu and Se) levels from serum samples, a previously described method was applied with minor modifications [25]. Microwave-assisted digestion was performed using a Milestone Ethos UP system (Milestone Srl, Sorisole, Italy). The 0.5-mL serum samples obtained by centrifugation were transferred to Teflon containers; then, 9 mL of nitric acid (HNO₃) and 1 mL of hydrogen peroxide (H₂O₂) were added, and digestion was performed in a microwave device at 150 °C for 120 min. The digested samples were diluted to a final volume of 15 mL with ultrapure water. The resulting solutions were measured using an Agilent 7800 ICPMS (Agilent Technologies, USA) instrument for trace element analysis. Standard solutions for Cu, Zn, and Se were prepared in 2% HNO₃, and indium, scandium, germanium, and bismuth were used as internal standards to ensure calibration accuracy. The ICP-MS system was operated with a radio frequency power of 1550 W; helium (He) gas flow was set at 4.3 mL/min, argon (Ar) plasma gas at 15 L/min, and auxiliary and carrier gases at 1.00 L/min and 0.99 L/min, respectively. The element concentrations obtained from the measurements were recorded in µg/L. All acids and reagents used in the analysis were of Suprapur grade (Merck, Germany).

The ICP-MS system utilized a chemically inert sample introduction configuration, comprising a glass MicroMist nebulizer, a quartz double-pass spray chamber, and a quartz torch. To maintain analytical precision, all quartz and nickel components were pre-cleaned by soaking overnight in 5–10% HNO₃, followed by thorough rinsing with ultrapure water and oven-drying. Before initiating measurements, the instrument underwent a 45-min helium gas purge and was calibrated using a tuning solution containing 1 µg/L of Ce, Co, Li, Mg, Tl, and Y. Each serum sample was analyzed in triplicate, and the acquired data were processed using Mass Hunter Workstation Software version 4.4 (Agilent 7800 ICP-MS, Top C.01.04).

The analytical performance of the method was evaluated using certified reference material (Seronorm™ Trace Elements Serum L-1, Norway). The method demonstrated high precision, with relative standard deviation (%RSD) values of 6.686% for Zn, 1.639% for Cu, and 2.898% for Se. The limit of detection (LOD) values were 0.467 µg/L for Zn, 0.028 µg/L for Cu, and 0.026 µg/L for Se, while the limit of quantification (LOQ) values were 1.557 µg/L, 0.093 µg/L, and 0.087 µg/L, respectively. These parameters confirm that the method was suitably sensitive and reproducible for trace element determination in serum samples [26].

### Biostatistical Analysis

Statistical analysis of the obtained data was performed using R Studio (v4.2.2) software. The distribution of each variable in both the patient and control groups was separately assessed using the Shapiro–Wilk test [27]. Variables with *p* > 0.05 in both groups were considered to exhibit a normal distribution, and an independent samples *t*-test was used for these variables. If *p* < 0.05 in at least one group, the Mann–Whitney *U* test was applied. In all analyses, the statistical significance threshold was set at *p* < 0.05.

To evaluate the diagnostic discriminative ability of variables showing significant differences between groups, ROC (Receiver Operating Characteristic) curve analysis was performed, and AUC (Area Under the Curve) values were calculated [28]. Spearman correlation analysis was conducted to assess relationships between variables [29], and the results were visualized using a heatmap.

Additionally, Principal Component Analysis (PCA) and Orthogonal Partial Least Squares Discriminant Analysis (OPLS-DA) were performed to evaluate sample-based variations and discriminatory patterns between groups [30]. Based on the OPLS-DA analysis, VIP (Variable Importance in Projection) scores were calculated to assess the contribution of variables to the model, and the top 15 most influential variables were identified.

### Machine Learning Approach

In this study, various machine learning algorithms were implemented using the Python programming language to identify variables that distinguish nasal polyp patients from healthy individuals and to evaluate classification performance [31]. The data were split into training (70%) and test (30%) sets, and all numerical variables were normalized using the StandardScaler method prior to analysis.

For classification purposes, logistic regression, naive Bayes, support vector machines (SVM), random forest, and K-nearest neighbors (KNN) algorithms were applied. The performance of these models was evaluated on the test data using metrics including accuracy, AUC (Area Under the Curve), precision, recall, F1-score, and confusion matrix. Following comparison, the logistic regression model demonstrating the highest classification performance was selected, upon which hyperparameter optimization (GridSearchCV) and fivefold cross-validation were performed [32].

Decision threshold optimization was conducted based on the model’s predicted probabilities, with the optimal classification boundary determined using the ROC curve and Youden’s index. Additionally, SHAP (SHapley Additive Explanations) analysis was applied to enhance interpretability of the model’s decision-making process and to demonstrate which variables contributed most to predicting the patient class [33]. The contributions of variables to the model were visualized and interpreted using summary plots.

## Results and Discussion

In the study, the normality of variables was assessed using the Shapiro–Wilk test. Based on this test, the Mann–Whitney *U* test was applied to parameters that did not follow a normal distribution (*p* < 0.05), while the independent samples *t*-test was used for normally distributed parameters (Table 1).

**Table 1:** Comparison of trace elements and biochemical parameters between patient and control groups, including normality test results and applied statistical tests

	Patient	Control	Shapiro–Wilk test *p* value	Applied test	*p*-value
Variable	Mean ± SD	Mean ± SD			
Age (unitless)	56.182 ± 12.644	40.632 ± 14.221	*p* > 0.05	*t*-test	0.001*
Se (µg/L)	512.860 ± 621.442	171.473 ± 219.436	*p* < 0.05	Mann–Whitney	0.126
Zn (µg/L)	2130.974 ± 3516.317	11,331.127 ± 27,697.378	*p* < 0.05	Mann–Whitney	0.018*
Cu (µg/L)	1938.912 ± 686.579	1102.018 ± 389.282	*p* < 0.05	Mann–Whitney	0.000*
Zn/Cu ratio (unitless)	0.972 ± 1.503	8.399 ± 18.166	*p* < 0.05	Mann–Whitney	0.000*
WBC (× 10⁹/L)	8.546 ± 2.152	6.965 ± 1.418	*p* > 0.05	*t*-test	0.009*
RBC (× 10^12^/L)	5.273 ± 0.542	5.028 ± 0.402	*p* > 0.05	*t*-test	0.115
HGB (g/dL)	14.945 ± 1.631	14.311 ± 1.667	*p* > 0.05	*t*-test	0.239
PLT (× 10⁹/L)	252.591 ± 48.408	289.000 ± 80.828	*p* < 0.05	Mann–Whitney	0.174
EO (%)	2.891 ± 1.899	3.268 ± 4.396	*p* < 0.05	Mann–Whitney	0.388
Glucose (mg/dL)	114.714 ± 40.030	84.938 ± 14.839	*p* < 0.05	Mann–Whitney	0.005*
Creatinine (mg/dL)	0.846 ± 0.177	0.722 ± 0.187	*p* > 0.05	*t*-test	0.056
ALT (U/L)	22.238 ± 9.273	21.138 ± 10.966	*p* < 0.05	Mann–Whitney	0.570
TSH (mIU/L)	1.609 ± 0.691	1.863 ± 1.270	*p* < 0.05	Mann–Whitney	0.856
NLR (unitless)	2.376 ± 1.084	1.813 ± 0.352	*p* > 0.05	*t*-test	0.033*
PLR (unitless)	110.050 ± 21.568	135.036 ± 39.746	*p* < 0.05	Mann–Whitney	0.016*

Values are presented as mean ± standard deviation. Units of each parameter are indicated in parentheses. Statistically significant differences (*p* < 0.05) are shown in bold and marked with an asterisk (*)

**Fig. 1:**
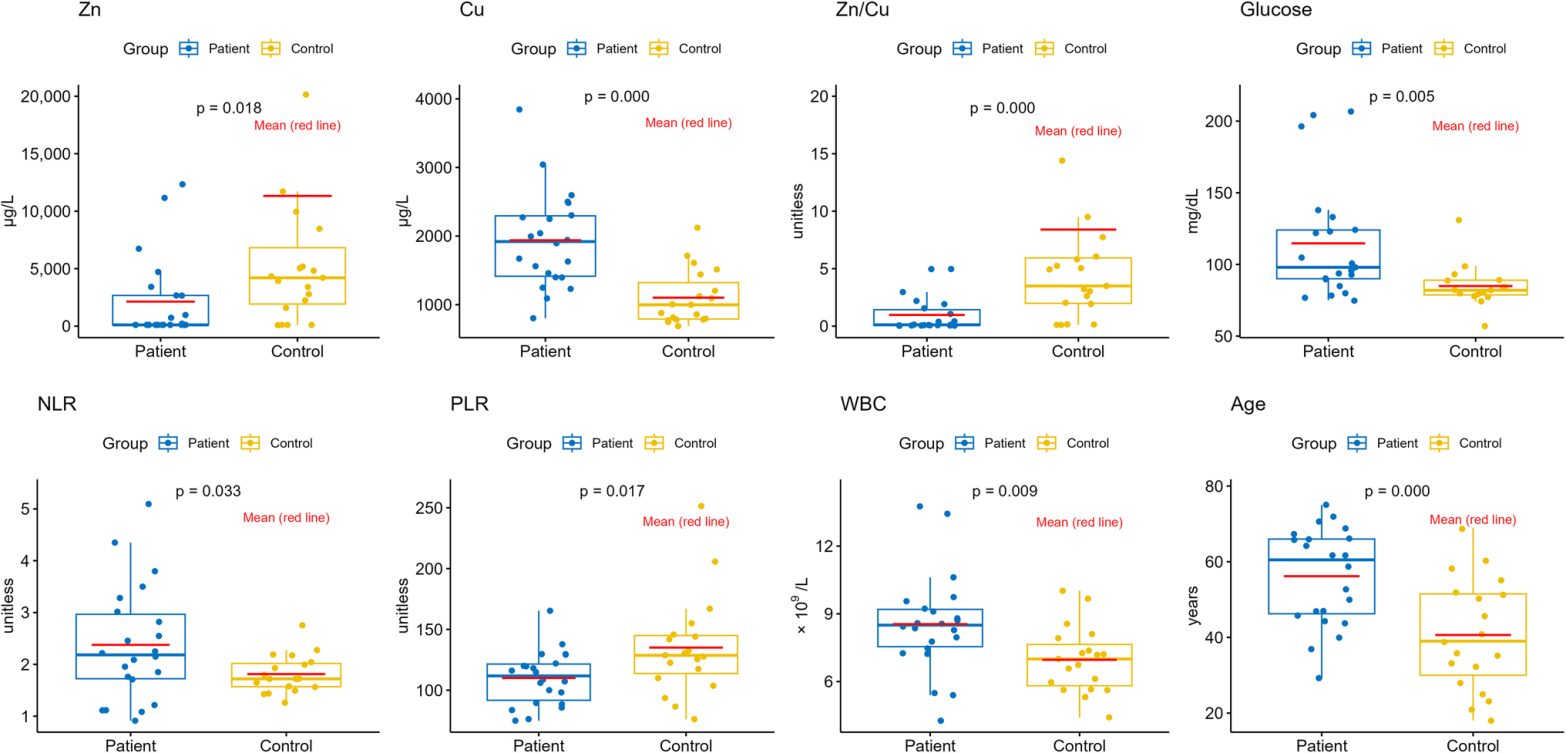
Box plots of parameters that show significant differences between groups

According to these test results, significant increases were observed in parameters such as WBC, Cu, glucose and NLR in the nasal polyp patient group, whereas PLR, Zn levels and the Zn/Cu ratio showed significant decreases (Fig. 1).

These results clearly demonstrate that nasal polyps are closely related to inflammation and oxidative stress processes. In particular, significant differences in WBC, NLR, and PLR values indicate that systemic inflammatory response plays an important role in the pathophysiology of nasal polyps. It has been reported in the literature that NLR and PLR are used as prognostic markers in many chronic inflammatory and autoimmune diseases [34]. The increase in these parameters may support that inflammation has reached a systemic level. In addition, the significant increase in serum Cu levels indicates that Cu contributes to proinflammatory processes and oxidative stress. The relationship between copper and chronic inflammation is also consistent with studies indicating that an increase in serum copper levels may be a marker of inflammation and oxidative stress [35, 36]. Therefore, it can be said that the increase in Cu levels can be considered as a reflection of the activation of inflammatory processes in nasal polyp patients. This aligns with findings from other inflammatory conditions where elevated copper levels are observed, suggesting a broader role for copper in systemic inflammatory responses beyond nasal polyps [37].

**Fig. 2:**
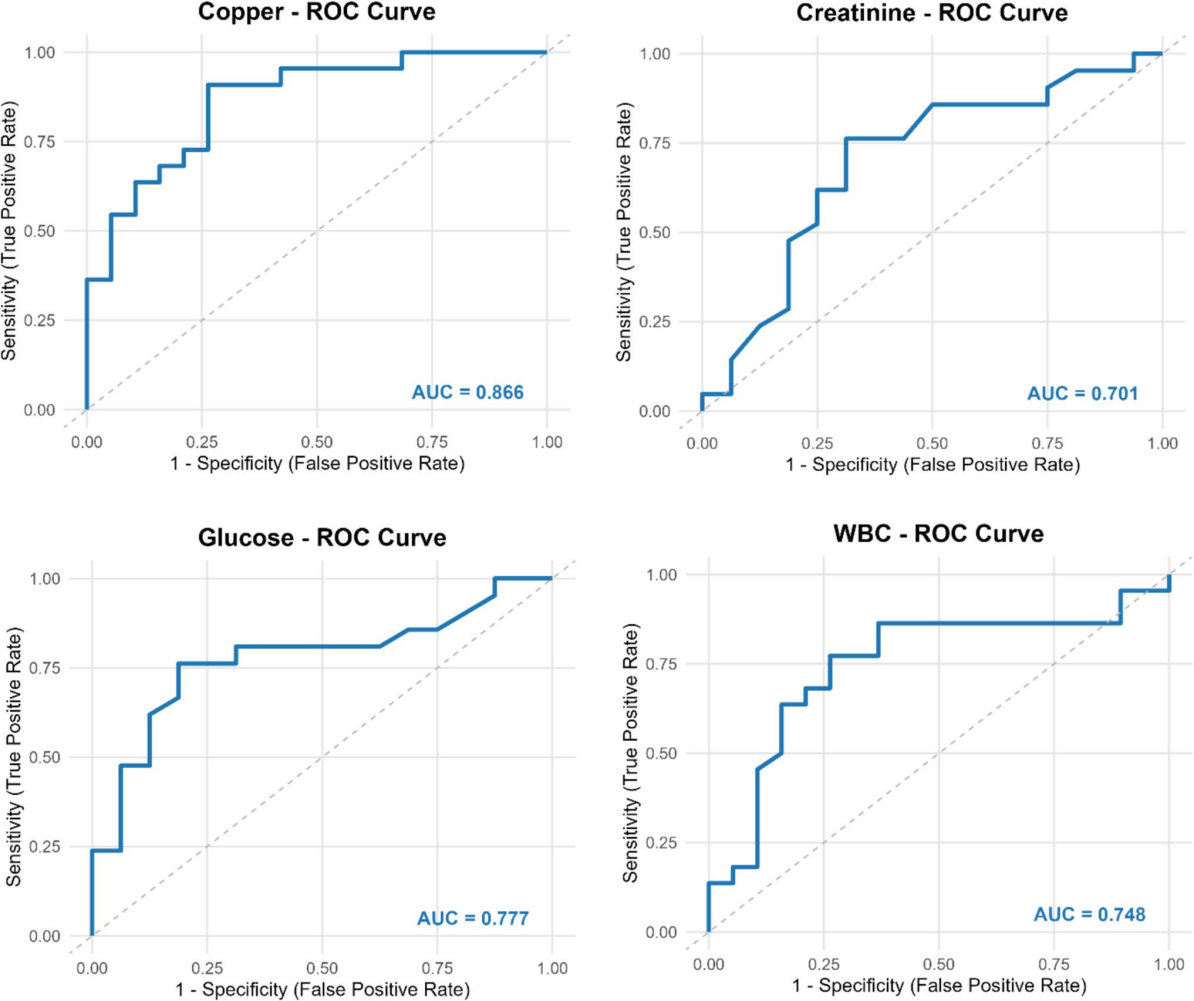
ROC curves for biochemical and trace elements in nasal polyps

Considering these results, ROC analysis was performed to reveal the biomarker potentials of the parameters that showed significant differences. As a result of this analysis, it was observed that Cu (AUC = 0.866), glucose (AUC = 0.777), WBC (AUC = 0.748) and creatinine (AUC = 0.701) had high discrimination power and biomarker potential (Fig. 2).

The high discrimination power of Cu (AUC = 0.866) reflects its strong relationship with inflammatory and oxidative processes in nasal polyps. Some previous studies on similar diseases have also indicated that copper may be a marker of inflammation [38]. Another noteworthy parameter was glucose (AUC = 0.777). In the study conducted by Wei Q. et al. [39], blood glucose levels in septic patients showed positive correlation with inflammatory response markers such as IL-6, TNF-α and IL-1β, and elevated blood glucose levels were associated with increased inflammatory responses. This finding explains glucose’s significant discriminatory power. Elevated leukocyte counts are widely recognized as a general marker of inflammatory processes in various clinical conditions and are known to be associated with chronic inflammation [40]. This finding explains the discriminatory power of WBC. The significant discriminative ability of creatinine levels (AUC = 0.701) suggests that metabolic processes related to kidney function may be affected in nasal polyps. The observed difference in serum creatinine levels indicates that inflammatory and metabolic burden may have subtle effects on renal function. Previous studies have demonstrated the impact of inflammation on kidney function and its association with alterations in creatinine levels [41]. However, we think that the clinical significance of this difference in creatinine levels should be confirmed by larger cohort studies.

**Fig. 3:**
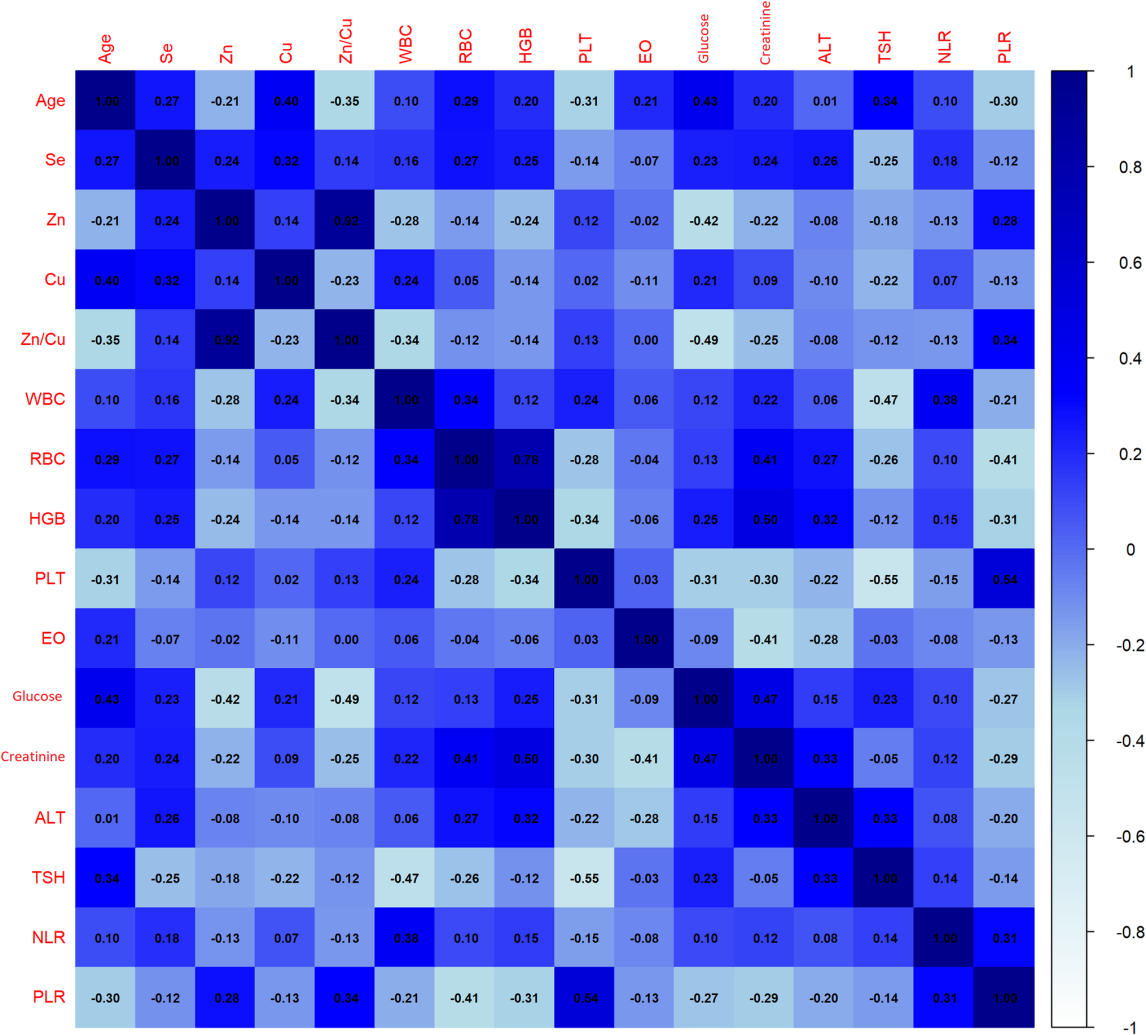
Spearman correlation heatmap of biochemical and trace element parameters

Then, Spearman correlation analysis was performed to observe the relationship between the variables and the correlation coefficients were visualized with a heat map (Fig. 3). As expected, a very strong positive correlation was observed between Zn and the Zn/Cu ratio (*r* = 0.92). Similarly, there was a high level of positive correlation between RBC and HGB levels (*r* = 0.78). On the other hand, significant negative correlations were found between Zn and glucose (*r* = –0.42) and the Zn/Cu ratio and glucose (*r* = –0.49). This may support the potential regulatory effect of Zn on glucose metabolism [42]. The negative correlation observed between PLT and NLR (*r* =  − 0.55) suggests that the balance between inflammation-related parameters may be disrupted. Positive correlations were found between age and glucose (*r* = 0.43) and creatinine (*r* = 0.20) levels. These findings indicate that metabolic changes due to aging may have an effect on the studied parameters [43].

These correlations, although revealing clear relationships between some parameters, show that there is no strong dependency between multiple variables. This suggests that variables can make independent contributions, especially in machine learning analyses.

**Fig. 4:**
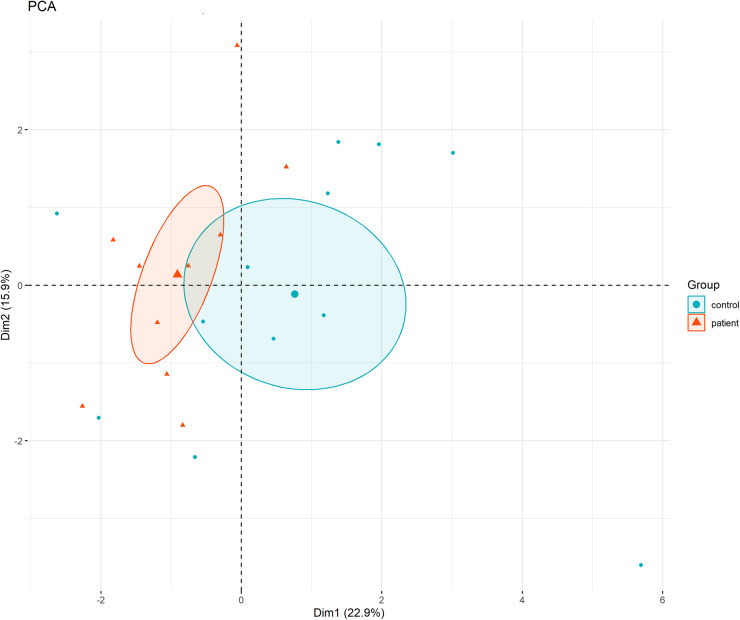
PCA distribution of patient and control groups

PCA analysis, which was performed to examine the general pattern in the data set and the distinction between the groups, showed that the patient and control groups were partially separated in some axes (Fig. 4). This indicates that there may be significant differences in the biochemical profiles of individuals. However, the partial overlap of the patient and control groups at some points in the PCA graph indicates that nasal polyps have a heterogeneous structure and the existence of subgroups specified in the literature [44]. It is thought that different stages of the disease or differences in inflammation severity may be the reason for this partial overlap in PCA analysis.

**Fig. 5:**
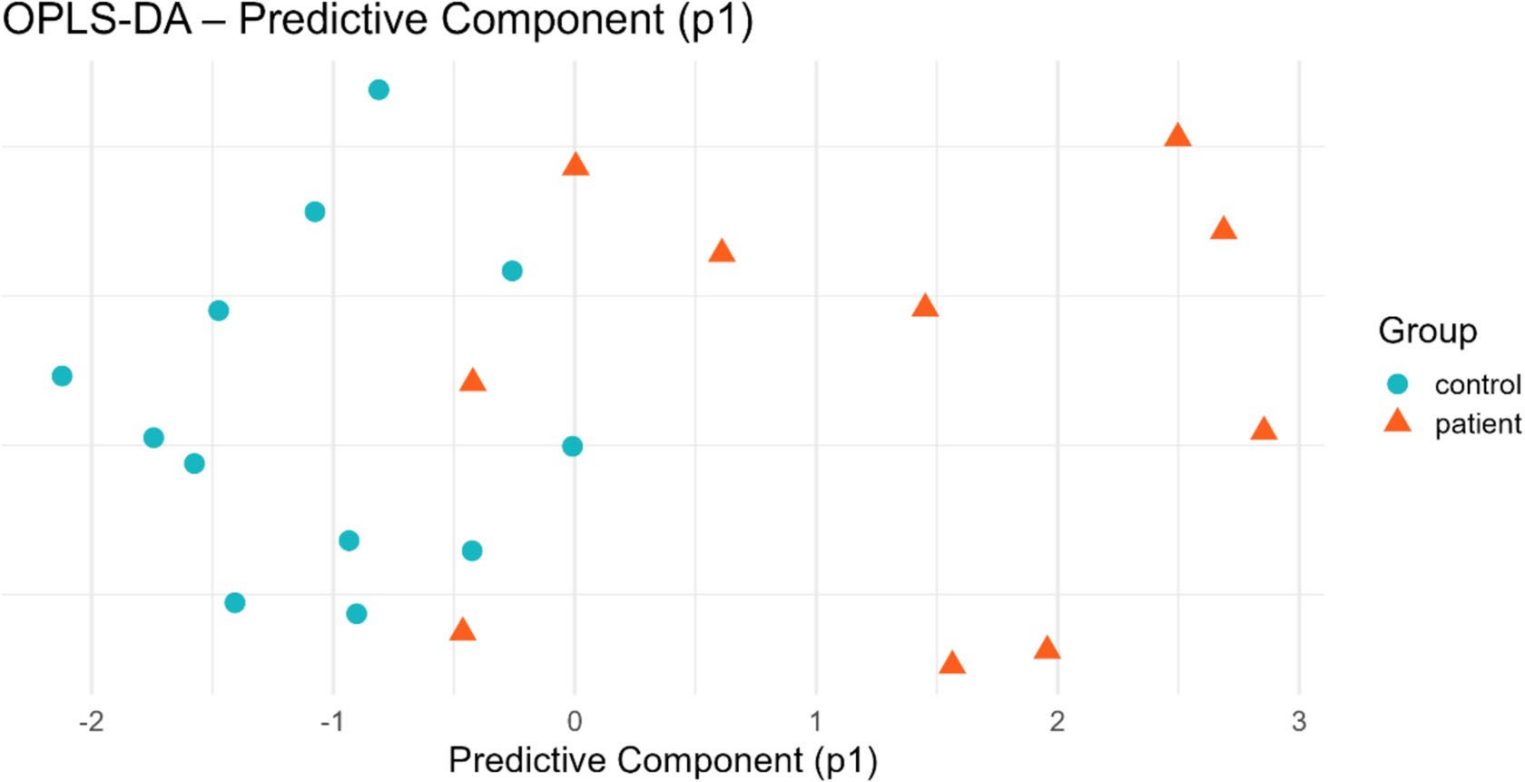
OPLS-DA score plot showing group separation

In the OPLS-DA analysis applied to better observe the classification performance and group separation, the groups were clearly separated (Fig. 5). This situation reveals that the selected parameters create strong and significant biochemical differences in characterizing nasal polyps.

**Fig. 6:**
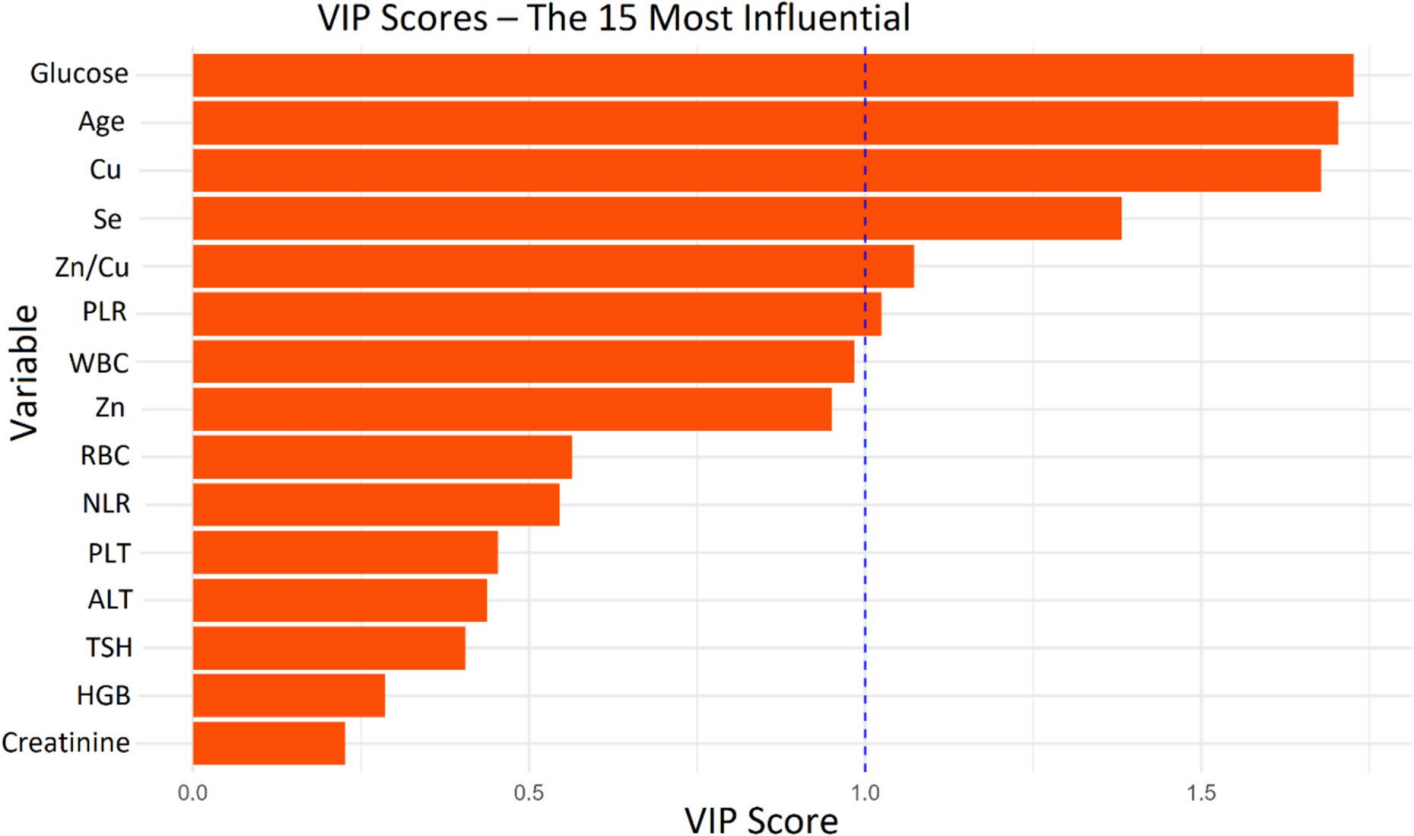
VIP scores highlighting the most discriminative variables

As a result of VIP scores calculated based on this model, variables such as Cu, Zn/Cu, glucose, Se and PLR were found to contribute highly to the model (Fig. 6). These variables were evaluated as the main biomarker candidates of the study from both statistical and biological perspectives. Zn is particularly striking from these results. Zn is an indispensable cofactor for more than 300 enzymes involved in metabolism and is also reported to play a role in aging, the immune system, apoptosis and oxidative stress [45]. Epidemiological and biological studies have shown that the imbalance between serum Zn and Cu levels is a causal factor for various diseases [46, 47]. This supports our findings. The high VIP score of PLR supports the importance of the systemic dimension of inflammation and the potential role of platelets in inflammatory processes. Considering that PLR is used as a prognostic indicator in many inflammatory diseases, it can be suggested that it may have a similar clinical value in nasal polyps [48, 49].

**Fig. 7:**
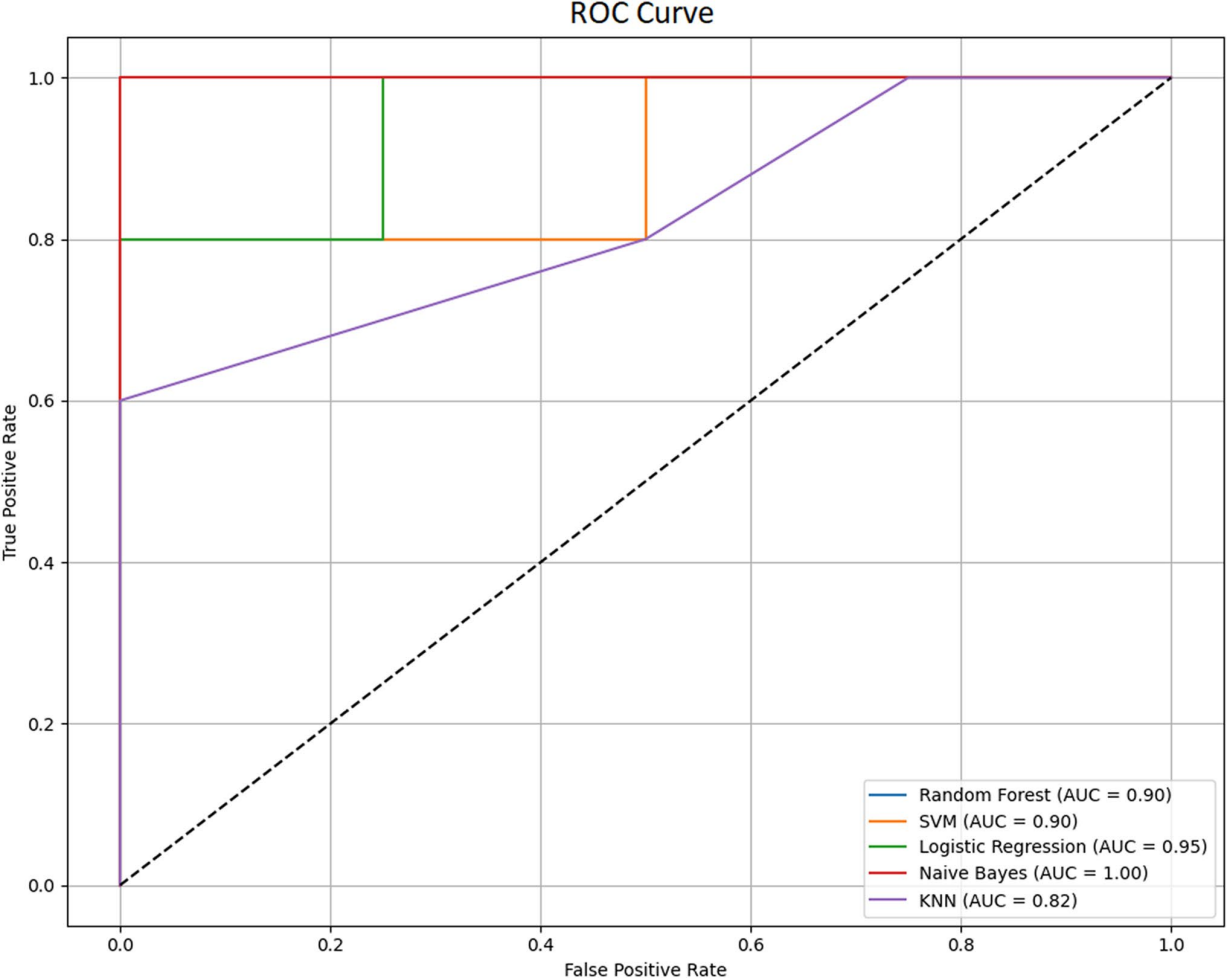
ROC curve comparison of machine learning models

To more thoroughly examine the contribution of prominent parameters identified through multivariate analyses to classification, machine learning methods were employed. In this context, missing data were first imputed, followed by scaling (normalization) using StandardScaler. Subsequently, Naive Bayes, SVM, Random Forest, KNN, and Logistic Regression models were trained with this processed data, and ROC curves were plotted for performance evaluation (Fig. 7).

**Fig. 8:**
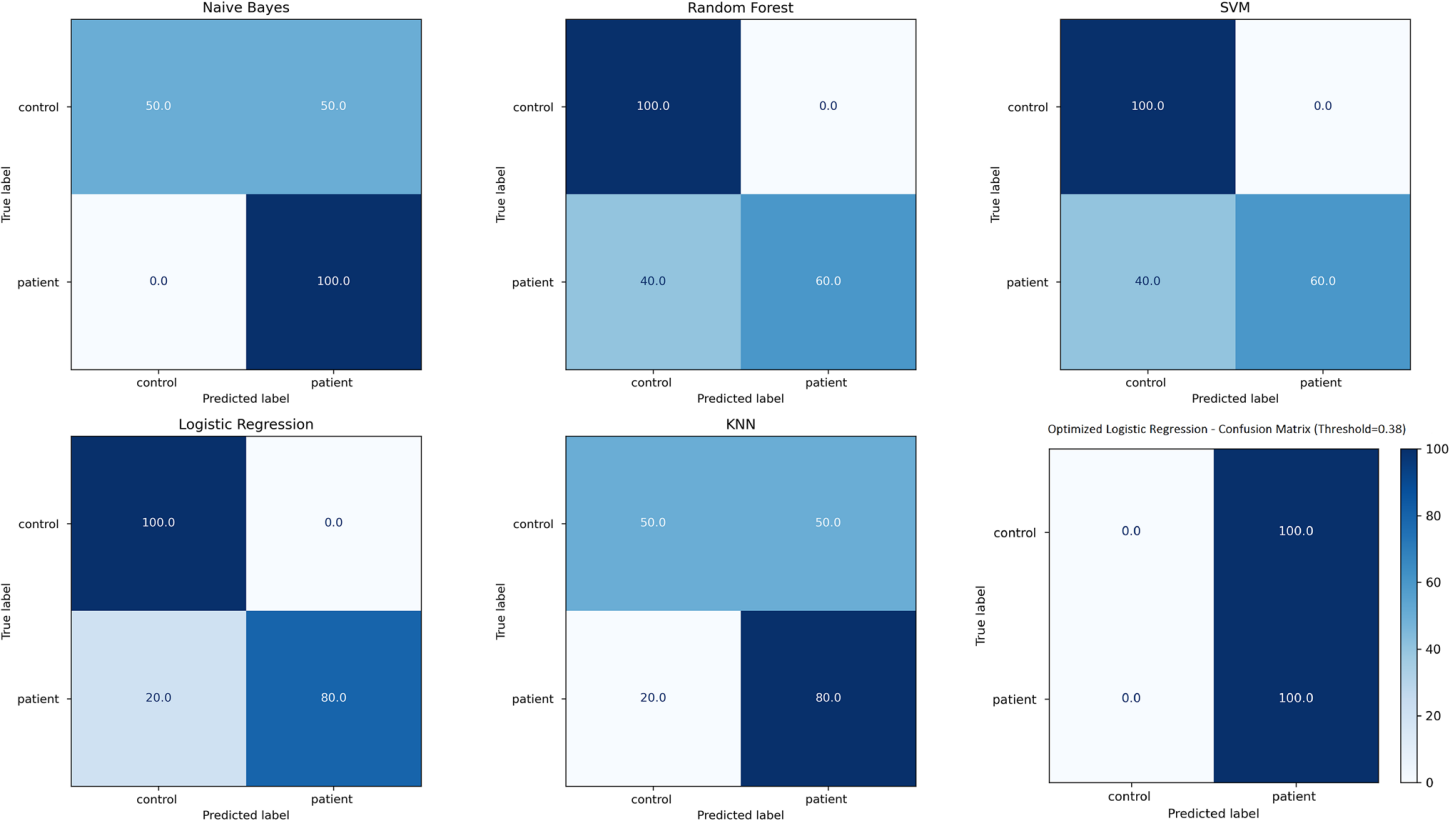
Confusion matrices of machine learning models for binary classification

While ROC curves reflect the classification performance of the models, the area under the curve (AUC) values represent the overall accuracy level of the models. ROC analysis results showed that Naive Bayes gave the highest AUC (AUC = 1) value, but this suggested that the model may be subject to over-learning [50]. An examination of the confusion matrix (Fig. 8) revealed that while the Naive Bayes model correctly predicted patients with 100% accuracy, it demonstrated poor performance in distinguishing healthy individuals (recall: 50%). Consequently, we proceeded with the Logistic Regression model, which yielded more balanced results. Analysis of this model’s confusion matrix showed 100% accuracy for healthy controls and 80% accuracy for patients.

Based on these findings, we performed hyperparameter optimization for the Logistic Regression model using GridSearchCV. To definitively determine whether the logistic regression model was overfitting, we conducted a fivefold cross-validation test. The results (mean AUC: 0.89, mean accuracy: 0.83) confirmed no evidence of overfitting [20]. Subsequently, threshold tuning was applied to adjust the decision boundary from 0.5 to 0.38. The reconfigured Logistic Regression model with this optimized threshold produced a confusion matrix that achieved 100% classification accuracy for both patient and healthy groups (Fig. 8).

These results clearly demonstrate the advantages of machine learning algorithms in the classification of biological data. It is observed that the biochemical parameters examined in nasal polyps, especially basic variables such as copper, glucose, and Zn/Cu ratio, have a very high discriminatory power in machine learning-supported classification. It has been reported in the literature that similar machine learning approaches can be used in clinical diagnosis and follow-up processes in various chronic inflammatory and metabolic diseases [21, 22]. The findings in this study similarly support the possibility of integrating these techniques into clinical practice in the diagnosis of nasal polyps.

**Fig. 9:**
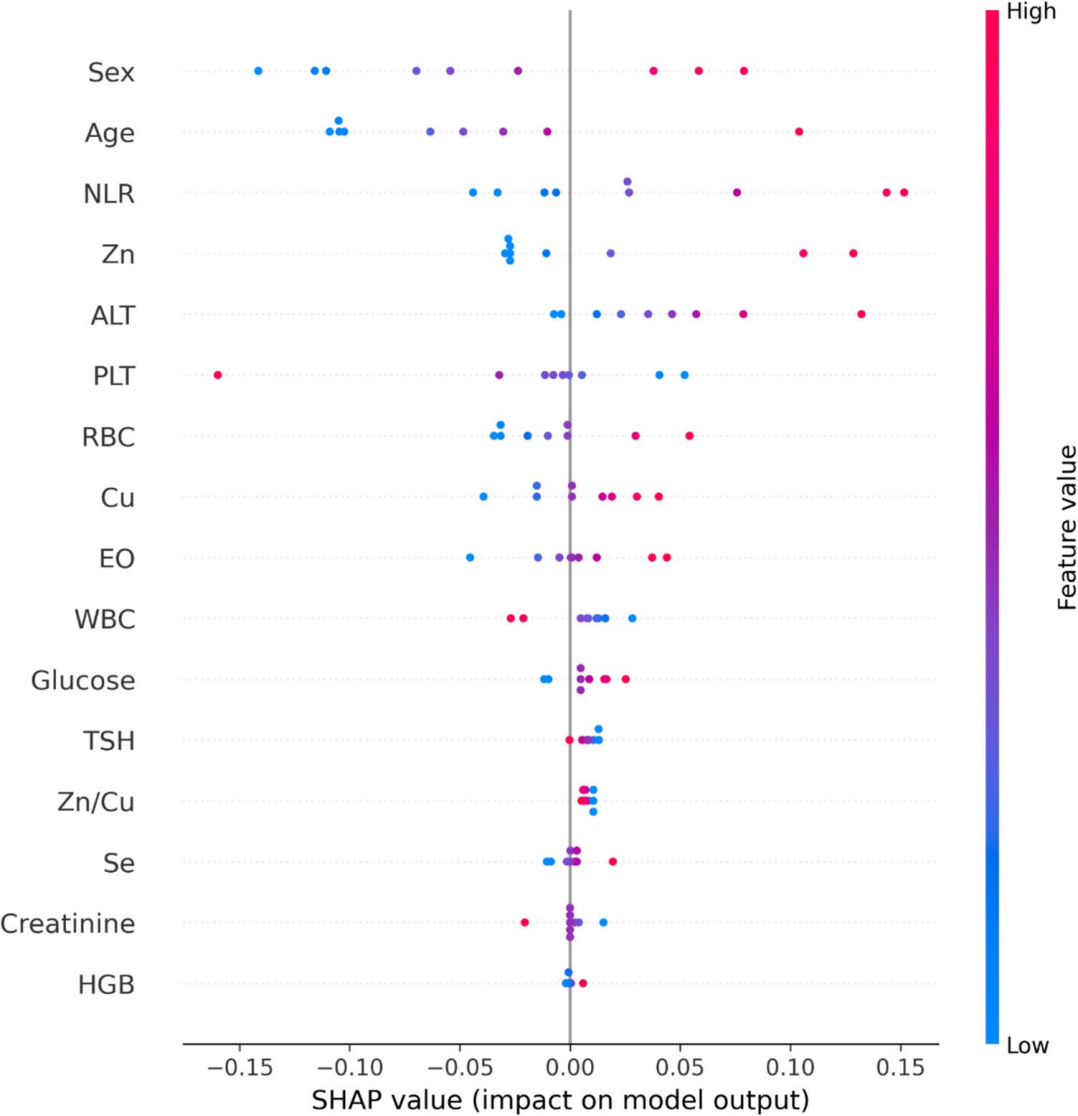
SHAP summary plot of feature contributions

In order to make the decision mechanism of the model more transparent and clinically interpretable, SHAP (SHapley Additive Explanations) analysis was performed and the variable effects were presented with a summary plot (Fig. 9).

The SHAP analysis enhanced the transparency of the decision-making process of the logistic regression model, thereby improving its clinical interpretability. According to the analysis results, parameters such as Zn, Zn/Cu ratio, Cu, and glucose were observed to have decisive effects within the model. While low Zn levels and a reduced Zn/Cu ratio positively influenced the patient classification, elevated Cu and glucose levels also strongly contributed to disease classification. These findings further confirm that Zn deficiency and a disrupted Zn/Cu ratio play a critical role in the development of nasal polyps and the inflammatory nature of the disease [51, 52]. The relationship between increases in serum Cu levels and inflammatory processes has been repeatedly emphasized in the literature, and the results in this study also support this relationship from a machine learning perspective [35]. These findings reveal that trace element imbalance and metabolic changes are effective in the pathogenesis of nasal polyps. In addition, the fact that parameters such as age and NLR are also effective in the model confirms the relationship between inflammation and aging [53, 54]. Since the increase in glucose levels also shows an effect towards the patient class, it can be said that this parameter has a critical biomarker potential in the diagnosis and follow-up of nasal polyps in terms of metabolic effects associated with inflammation. This result in the SHAP analysis is also consistent with the high glucose discrimination finding in the ROC analysis [55]. As a result, the SHAP method has been evaluated as a powerful tool that facilitates the integration of biomarkers into clinical diagnostic processes. Although age was significantly different between the NP group and the control group (56.2 ± 12.6 vs. 40.6 ± 14.2 years, *p* = 0.001), we carefully examined its potential confounding effect on the observed results. Age was included as an independent variable in both multivariate and machine learning analyses. Notably, SHAP analysis demonstrated that age contributed modestly to model predictions but did not dominate the classification outcome. Parameters such as Zn, Cu, Zn/Cu ratio, and glucose exhibited higher SHAP values, indicating stronger predictive power than age. These findings suggest that while age may have some influence, it does not fully account for the biochemical differences observed between groups. Nevertheless, age-related effects should be considered in future studies with larger, age-matched cohorts to ensure robust generalization of these results.

Validation of these findings in larger and more diverse cohorts is essential before clinical implementation. Future studies that more precisely characterize the biochemical profiles of nasal polyps could support the development of improved diagnostic and monitoring strategies.

While this study offers important insights, several limitations must be acknowledged. The small sample size and single-center design may limit the generalizability of the results. Additionally, the restricted panel of biochemical and trace element markers may not capture the full complexity of nasal polyp pathogenesis. The cross-sectional design precludes causal inference, and potential selection or confounding biases cannot be entirely excluded. Prospective and longitudinal studies are needed to assess the temporal dynamics and predictive value of the identified markers.

Building on these findings, future research should investigate the mechanistic roles of trace element imbalances, particularly zinc and copper, in inflammation and oxidative stress pathways. Incorporating multi-omics approaches (e.g., proteomics, metabolomics), exploring gene–environment interactions, and validating biomarker panels in clinical trials may help realize personalized diagnostic and therapeutic strategies for nasal polyp management.

## Conclusion

In this exploratory study, we comprehensively evaluated serum trace element levels and biochemical parameters in patients with nasal polyps using classical statistical methods, multivariate analysis, and machine learning approaches. Statistically significant differences were observed, including increased levels of Cu, glucose, WBC, NLR, and PLR, as well as decreased Zn levels and Zn/Cu ratios, suggesting a potential association with underlying inflammatory and oxidative processes.

The logistic regression model, combined with SHAP analysis, identified Zn, Cu, the Zn/Cu ratio, and glucose as the most influential variables for distinguishing between patient and control groups, providing both predictive utility and enhanced interpretability.

However, due to the limited sample size and single-center design, these results should be interpreted with caution. Validation in larger, independent cohorts is necessary before these biomarkers can be considered for clinical implementation. Nevertheless, our findings provide a basis for future investigations into the diagnostic and pathophysiological significance of trace element imbalance in nasal polyps.

## Data Availability

Data will be made available on request.

## Declarations

### Use of Large Language Models, AI and Machine Learning Tools

None declared.
